# Decidual macrophage subsets and polarization puzzle during the human early pregnancy

**DOI:** 10.3389/fimmu.2025.1610891

**Published:** 2025-07-14

**Authors:** Huiling Liu, Liping Zhang

**Affiliations:** Department of Obstetrics, Huai’an Maternal and Child Health Care Hospital affiliated to Yangzhou University, Huai’an, Jiangsu, China

**Keywords:** decidual macrophages, M1-like dMφs, M2-like dMφs, human early pregnancy, polarization

## Abstract

At the maternal-fetal interface from human early pregnancy, decidual macrophages (dMφs) comprise approximately 20% of the leukocyte population, displaying a distinct immunophenotype characterized by hybrid functional features that transcend conventional M1/M2 polarization paradigms. The dynamic balance between M1-like dMφs and M2-like dMφs in human early pregnancy is closely related to the success of pregnancy. However, the comprehensive subsets profiling of dMφs and the factors influencing polarization haven’t been elucidated until recent years. In this review, we first delineate the dMφs compositional proportion and subsets profiling during early gestation. Second, we clarify the mechanisms underlying dMφs recruitment and tissue residency. Finally, we comprehensively synthesize molecular drivers of dMφs polarization and the functional specialization of polarized dMφs in sustaining successful pregnancy. A comprehensive understanding of the molecular network governing dMφs polarization dynamics and their functional contributions to gestational processes will provide crucial insights for developing targeted therapeutic strategies to address pregnancy-related complications.

## 1 Introduction

During early human pregnancy, the uterine mucosa undergoes a specialized transformation into the decidua, a receptive tissue that facilitates the implantation of fetal-derived trophoblast cells. This critical biological process initiates a cascade of gestational adaptations, including extensive remodeling of uterine smooth muscle cells and spiral arteries. These coordinated morphological changes ultimately culminate in the establishment of a functional placental organ. Within the placental microenvironment, invasive trophoblasts, decidual stromal cells (DSCs), and specialized immune populations form direct interaction (1). The dynamic crosstalk among these cellular components is essential for maintaining maternal-fetal immune tolerance and ensuring gestational success. Notably, placental macrophages (Mφs), which exhibit distinct phenotypic characteristics compared to their other tissue counterparts, emerge as central regulators in human early pregnancy.

Mφs are generally categorized M1 (classically activated) and M2 (alternatively activated) subtypes (2). M1 Mφs function as pro-inflammatory immune effector characterized by three distinct features: (1) elevated expression of antigen-presenting molecules (MHC-II) and co-stimulatory molecules (CD80, CD86) (3); (2) increased secretion of pro-inflammatory cytokines (interferon-gamma (IFN-γ), reactive oxygen species (ROS), interleukin-12 (IL-12), IL-23, IL-1β); (3) metabolic reprogramming toward glycolysis with concomitant ROS generation (4–6). In contrast, M2 Mφs demonstrate immunosuppressive properties through three complementary mechanisms : (1) immunoregulatory mediator production including IL-10 and transforming growth factor beta (TGF-β); (2) up-regulation of surface marker scavenging receptors and mannose receptor (CD206, CD209, CD163) (7, 8); (3) metabolic shift toward oxidative phosphorylation (OXPHOS) coupled with fatty acid β-oxidation (6). This unique combination enables M2 Mφs to perform tissue-protective functions such as apoptotic cell clearance, extracellular matrix remodeling, and resolution of inflammatory responses.

This functional dichotomy between M1/M2 Mφs is governed by distinct activation pathways. M1 Mφs are typically activated through exposure to pro-inflammatory mediators including tumor necrosis factor-alpha (TNF-α) and IFN-γ, or *via* engagement of pathogen-associated molecular patterns (PAMPs) such as bacterial lipopolysaccharide (LPS) (9). In contrast, M2 Mφs polarization is orchestrated by anti-inflammatory cytokines, notably IL-4 and IL-13 (8). Pro-inflammatory mediators mediate M1 polarization through engagement of surface receptors, including cytokine receptors and pattern recognition receptors such as toll-like receptor 4 (TLR4). This signaling cascade activates transcription factors like nuclear factor-kappa B (NF-κB) and signal transducer and activator of transcription 1 (STAT1), which drive the expression of genes characteristic of the pro-inflammatory M1 phenotype (10). Conversely, anti-inflammatory cytokines stimulate transcription factors such as STAT6 and peroxisome proliferator-activated receptors (PPARs), facilitating the transcriptional program associated with the immunoregulatory M2 phenotype (11).

Placental Mφs consist of maternal-derived decidual Mφs (dMφs) and fetal-derived Hofbauer cells. Recent years, placental Mφs have become a research hotspot. Hofbauer cells closely resemble alternatively activated M2 Mφs (12), while dMφs display dynamic plasticity and functional heterogeneity that diverge from the classical M1/M2 dichotomy (13). Therefore, in this review, we mainly focus on maternal-derived dMφs. In response to this evolving understanding, the scientific community is increasingly adopting the M1-like and M2-like dMφs. M1-like dMφs and M2-like dMφs denote a broader spectrum of Mφ status that may overlap or transition between these traditional M1 and M2 Mφ categories. The immune status of dMφs is suggested to be dynamic during gestation, with an M1-like status during the peri-implantation period, a mixed M1/M2-like status during early pregnancy followed by an M2-like status during the second trimester, and an M1-like status by the end of pregnancy (3, 14–16). In human early pregnancies, the dynamic balance between M1-like dMφs and M2-like dMφs is closely related to the the success of pregnancy. M1-like dMφs initiate local inflammation and aid embryo implantation and decidualization. M2-like dMφs maintain immune tolerance, phagocytose apoptotic cells and participate in spiral artery remodeling. However, a disruption of the balance of dMφs may result in various adverse pregnancy outcomes including recurrent spontaneous abortion (RSA), pre-eclampsia (PE) and fetal growth restriction (FGR). Only by fully understanding the factors regulating dMφs polarization and roles of dMφs in pregnancy will we be able to develop interventions for the treatment of these various pregnancy complications. Therefore, this systematic review delineates the subsets characteristics of polarized dMφs, mechanisms underlying dMφs recruitment, and molecular drivers of dMφs polarization at the human maternal-fetal interface during early gestation.

## 2 The dMφs frequency at the maternal-fetal interface

In the human first-trimester pregnancy, the most preponderant maternal immune cells at the maternal-fetal interface are CD56^+^decidual natural killer (dNK) cells, which account for approximately 60%, and then followed by dMφs at 20% and T cells at 10% (17–19). These findings were typically derived from single-cell suspension techniques. Notably, the inevitable loss of specific cell populations during the isolation procedure may potentially compromise the accuracy of the results. Krop et al. conducted a comparative analysis of immune cell frequencies in the human decidua between tissue sections and single-cell suspensions (20). Their findings revealed significantly higher myeloid cell proportions in tissue sections (35.8%, 52.5%, and 60% during the first, second, and third trimesters, respectively) compared to single-cell suspensions (20%, 26.8%, and 9.4% at corresponding gestational stages) (20). Complementing these findings, a multi-omics study integrating spatial proteomics and transcriptomics demonstrated dynamic shifts in decidual immune composition: while dNK cells predominated at 6 weeks of gestation, dMφs progressively increased from 8 weeks, surpassing dNK cells by 12 weeks (21). Collectively, these results demonstrate that dMφs—the principal antigen-presenting cells (APC) in the human decidua—were substantially underrepresented in conventional analytical approaches, highlighting methodological limitations in assessing their true physiological prevalence.

## 3 The characteristics of dMφs subsets in human early pregnancy

### 3.1 Traditional classification of dMφs subsets

In recent years, several investigators had performed single-cell analysis of human decidual immune cells, either by RNA sequencing or flow cytometric cell sorting. This had led to a more detailed insight into the different M1/M2-like polarized dMφs encountered at the human decidua. The phenotypic and functional heterogeneity of dMφs have been characterized through surface marker profiling including phagocytic receptor CD209(DC-SIGN), intercellular adhesion molecule-3 (ICAM-3), T-cell immunoglobulin mucin-3 (Tim-3), cyclooxygenase-2 (COX-2), chemokine (CC motif) receptor 1 (CCR1), CCR2, indoleamine 2, 3-dioxygenase 1 (IDO1), CD36, receptor activator of nuclear factor kappa B (RANK) and CD11c (**Table 1**).

**Table 1:** Traditional classification and characteristics of dMφs in human early pregnancy.

Subset	Proportion	Key markers	Other features	Polarization outcome	Reference
CD209^+^dMφs	~36.9%	CD209^+^	Immature dendritic-like; efficient antigen uptake; no T cell activation; interacting with ICAM-3^+^LGLs	M2-like	(22)
ICAM-3^-^dMφs	~60%	ICAM-3^-^, CD163^+^, CD206^+^, CD209^+^, NRP-1^+^	Enhanced M2 polarization	M2-like	(23)
Tim-3^+^dMφs	~23%	Tim-3^+^, CD163^+^, CD206^+^, CD209^+^, CD80^+^, CD86^+^	Higher production of angiogenic growth factors (including PDGF-AA, TGF-α, and VEGF); inducing Th2/Treg bias	M2-like	(25, 26)
CCR1^+^dMφs	~59.98%	CCR1^+^, CD163^+^, CD206^+^, TGF-β^+^, IL-10^+^	Tissue remodeling; immunosuppressive (↑CD163, CD206, IL-10, TGF-β; ↓CD80, CD86)	M2-like	(29)
COX-2^+^dMφs	~36%	COX-2^+^, CD163^+^, CD206^+^, CD209^+^, IDO1^+^	Suppressed IFN-γ, IL-23, IRF4	M2-like	(31)
IDO^+^dMφs	~34.08%	IDO^+^, CD206^+^, CD209^+^, CD163^+^	Down-regulated in RSA(14.6%); lower CD86; promoting EVT proliferation and invasion	M2-like	(32)
CD36^+^dMφs	~16.5%	CD36^+^, IL-6^+^, IL-1β^+^, TNF-α^+^, IFN-γ^+^	Pro-inflammatory cytokine profile; associated with miscarriage(38.6%)	M1-like	(34)
RANK^+^dMφs	~86.4%	RANK^+^, CD163^+^, CD206^+^, CD209^+^, IL-10^+^	M2-like identity reinforced by RANKL stimulation	M2-like	(35)
CD11c-based subsets					
CD11c^hi^dMφs	~20%	CD11c^hi^, IL-10^hi^,CD206^lo^, CD209^lo^	Lipid metabolism; IL-10 dominance, mixed cytokines	Mixed M1/M2	(13)
CD11c^lo^dMφs	~69%	CD11c^lo^, IL-10^lo^, CD206^hi^, CD209^hi^	High level phagocytic receptors; tissue-remodeling transcripts, mixed cytokines	Mixed M1/M2	
CD11c/CCR2-based subsets					
CCR2^+^CD11c^hi^	~15%	CCR2^+^, IL-1β^+^, COX-2^+^, lysozyme C^+^	Pro-inflammatory;high phagocytosis; proximal to EVTs,	M1-like	(36)
CCR2^-^CD11c^hi^	~5%	CCR2^-^, CD209^lo^, HMOX1^+^	anti-inflammatory, maximal phagocytosis; reduced CD209; proximal to EVTs,;	M2-like	
CCR2^+^CD11c^lo^	~80%	CD209^hi^	minimal phagocytosis; high CD209; widespread in the decidua	/	

LGLs, large granular lymphocytes; RSA, recurrent spontaneous abortion; EVT, extravillus trophoblast; HMOX1, heme oxygenase 1; Tim-3, T-cell immunoglobulin mucin-3; PDGF, platelet-derived growth factor; TGF-α, transforming growth factor α.

Pioneering work by Kammerer et al. revealed unique properties of dMφs in human early pregnancy (22). CD209 is a well-known marker for classic M2 Mφs. Compared to endometrial Mφs, 36.9% of dMφs specifically expressed the phagocytic receptor CD209, exhibiting an immature dendritic cell-like phenotype (**Table 1**). These CD209^+^dMφs demonstrated efficient antigen uptake capacity *in vitro* but failed to stimulate *naïve* allogeneic T cells.

Further stratification based on ICAM-3, Tim-3, CCR1, COX-2, IDO-1 and CD36 expression revealed functional divergence. ICAM-3, a transmembrane glycoprotein mediating leukocyte adhesion and cellular survival, has not been definitively classified as a marker for either canonical M1 or M2 Mφs subsets. Intriguingly, about 60% of human early pregnancy dMφs lacked ICAM-3 (23) (**Table 1**). Notably, compared with ICAM-3^+^dMφs, ICAM-3^-^dMφs displayed enhanced M2 polarization, with significantly elevated CD163, CD206, CD209, and neuropilin-1 (NRP-1) (23) (**Table 1**). This inverse correlation between ICAM-3 expression and M2 marker profiles positioned ICAM-3 as a potential identifier of pro-inflammatory M1-like dMφs at the maternal-fetal interface. Tim-3, a checkpoint receptor expressed by a wide variety of immune cells, exerts anti-inflammatory effects through suppression of ROS generation and inflammasome-dependent cytokine secretion (IL-1β, IL-18) in Mφs (24). Strikingly, at the maternal-fetal interface during human early pregnancy, Tim-3^+^dMφs demonstrated dual functional specialization: (1) pro-angiogenic capacity: enhanced production of growth factors including platelet-derived growth factor-AA (PDGF-AA), TGF-α, and vascular endothelial growth factor (VEGF); (2) immunomodulatory activity: promoting Th2 and Treg bias (25, 26) (**Table 1**). The functional dynamics of CCR1 in Mφs regulation demonstrated complex tissue-specific and ligand-dependent characteristics (27, 28). Recent study revealed distinct anti-inflammatory properties of CCR1^+^dMφs in human early pregnancy. Compared to their CCR1^-^ counterparts, CCR1^+^dMφs displayed elevated expression of scavenger receptors (CD163, CD206), enhanced production of immunoregulatory cytokines (IL-10, TGF-β) (29) (**Table 1**). The COX-2/PGE2 axis, traditionally associated with M1 polarization (30), exhibited paradoxical regulatory effects in human dMφs. COX-2^+^dMφs paradoxically exhibited higher levels of CD163, CD206, CD209 and IDO-1, as well as lower levels of interferon regulatory factor 4 (IRF4), IFN-γ and IL-23 than COX-2^-^dMφs (**Table 1**), suggesting that COX-2^+^dMφs presented an M2-like phenotype (31). The immunomodulatory enzyme IDO, primarily expressed by APC including Mφs, mediates tryptophan catabolism *via* the kynurenine pathway. The percentage of IDO^+^dMφs from women with normal pregnancy and RSA were 34.08% and 14.6%, respectively (32). IDO^+^dMφs had higher levels of CD206, CD209 and CD163, and a lower level of CD86 compared with IDO^−^dMφ (**Table 1**), suggesting that IDO^+^dMφs displayed an M2-like phenotype during human early pregnancy (32). CD36, a multifunctional receptor mediating lipoprotein recognition, apoptotic cell clearance, and fatty acid transport, also serves as a pattern recognition receptor. Within the classical M1/M2 polarization framework, CD36 demonstrated preferential association with M2 Mφs through lipid-mediated mechanisms (33). Mechanistically, CD36-dependent triglyceride trafficking facilitated up-regulation of canonical M2 markers (CD206, CD163) (33). Paradoxically, CD36 presented strong link with M1-like dMφs in human early pregnancy (34). Clinical analyses revealed a striking elevation in CD36^+^dMφs prevalence among RSA patients (38.6% versus 16.5% in normal pregnancies) (**Table 1**). These CD36^+^dMφs exhibited amplified pro-inflammatory cytokines (IL-1β, IL-6, TNF-α, IFN-γ) compared to their CD36^-^ counterparts, with comparative analysis showing further up-regulation of pro-inflammatory cytokine in RSA-derived CD36^+^dMφs relative to normal pregnancy controls (34). This tissue-specific inversion of CD36’s polarization association suggested microenvironment-driven functional plasticity. The molecular mechanisms underlying this phenomenon was systematically clarified in Section 5.

The RANK/RANK ligand (RANKL) axis emerged as another modulator of dMφs plasticity in human early pregnancy. RANK^+^dMφs, 86.4% at human dMφs from early pregnancy, exhibited up-regulated M2 markers (CD206, CD209, CD163, IL-10) compared with RANK^-^dMφs (**Table 1**) (35). RANKL stimulation markedly enhanced the M2 characteristics while suppressing M1 features in RANK^+^dMφs, demonstrating the RANK/RANKL axis’s pivotal role in controlling dMφs polarization (35).

The CD11c-based classification revealed hybrid phenotype. CD11c^hi^dMφs (20%) showed IL-10 dominance, low phagocytic receptors (CD206, CD209) and lipid metabolism gene enrichment, while CD11c^lo^dMφs (69%) expressed higher level of CD206, CD209 and tissue-remodeling transcripts as well as low IL-10 (13) (**Table 1**). Both populations secreted mixed cytokines, reflecting a mixed M1/M2-like dMφs states (13). Further CCR2 stratification of CD11c subsets identified functional gradations (36) (**Table 1**). CCR2^+^CD11c^hi^dMφs (15%), proximal to EVTs, co-expressed pro-inflammatory mediators (IL-1β, COX-2, lysozyme C) with high phagocytic capacity (36). CCR2^-^CD11c^hi^dMφs (5%) exhibited maximal phagocytosis but reduced CD209, a characteristic feature that is also associated with the CD11c^hi^dMφs (36). CCR2^-^CD11c^hi^dMφs, also proximal to EVTs, expressed higher levels of heme metabolism genes indicating its anti-inflammatory role (36). CCR2^-^CD11c^lo^ (80%), widespread in the decidua, showed minimal phagocytic activity and high CD209 correlating well with CD11c^lo^dMφs subset (36). Due to the complexity of dMφs, Ning et al. proposed that the function of dMφs in tissue remodeling versus inflammation will not be easily attributable to one or other subset (16).

### 3.2 Emerging multi-dimensional classification of dMφ subsets

While traditional classification of dMφs relies on one or two surface markers, recent advances in spatial multi-omics and single-cell technologies unveiled a far more complex landscape, emphasizing the necessity of multi-parameter stratification. Spatial proteomic/transcriptomic studies resolved CD209^+^dMφs (77% prevalence in early pregnancy) into three functionally distinct subsets:Mac2a (CD11c^-^HLA-DR^+^), Mac2b (CD11c^+^HLA-DR^+^) and Mac2c (CD11c^-^HLA-DR^-^) (21) (**Table 2**). CD209^–^dMφs were subclustered on the basis of CD68 expression: Mac1a (CD68^+^) and Mac1b (CD68^-^) (21). Imaging mass cytometry (IMC) have further resolved the heterogeneity of dMφs, identifying six distinct subclusters during early pregnancy. Among these, four subclusters were definitively stratified by combinatorial expression of HLA-DR and CD209: dMφ1 (HLA-DR^-^CD209^+^), dMφ2 (HLA-DR^+^CD209^+^), dMφ4 (HLA-DR^-^CD209^-^) and dMφ5 (HLA-DR^+^CD209^-^) (20) (**Table 2**). Notably, dMφ1 and dMφ4—both lacking HLA-DR expression—constituted the dominant populations in first-trimester decidua, suggesting their potential roles in early gestational immune modulation. A novel hybrid subset dMφ3 exhibited dual expression of myeloid markers (CD14, CD68) and NK cell markers (CD56), a phenotype previously uncharacterized in decidual tissue (20). This unique co-expression pattern hinting its trans-differentiation potential.

**Table 2:** Multi-dimensional classification of dMφ subsets.

Spatial multi-omics classification					
Subset	Proportion	Key markers	Other features	Polarization outcome	Reference
Mac2a	~77%	CD209^+^, CD11c^-^, HLA-DR^+^	/	/	(21)
Mac2b		CD209^+^,CD11c^+^, HLA-DR^+^	/	/	
Mac2c		CD209^+^, CD11c^-^, HLA-DR^-^	/	/	
Mac1a	N/A	CD209^-^, CD68 ^+^	/	/	
Mac1b	N/A	CD209^-^, CD68 ^-^	/	/	
Imaging mass cytometry-defined subclusters					
dMφ1	~28%	HLA-DR^-^, CD209^+^	/	/	(20)
dMφ2	~4%	HLA-DR^+^, CD209^+^	/	/	
dMφ3 (hybrid)	~8%	CD14^+^, CD68^+^, CD56^+^, CD16^+^	/	/	
dMφ4	~52%	HLA-DR^-^, CD209^-^	/	/	
dMφ5	~5%	HLA-DR^+^, CD209^-^	/	/	
Tissue-resident subsets					
decBAMs	N/A	CD163^+^, CD206^+^, CD11c^hi^, HLA-DR^1o^	Secreting IL-10, VEGFA, HMOX1; promoting Treg induction	M2-like	(37)
decPAMs	N/A	CD163^+^, CD206^+^, CD11c^1o^, HLA-DR^+^	APC-like activity; maintaining immune surveillance in non-invasion zones	M1-like	

decBAMs, decidua basalis-associated macrophages; decPAMs, decidua parietalis-associated macrophages; APC, antigen presenting cell; HMOX, heme oxygenase 1.

### 3.3 Spatial classification of dMφs subsets

The dMφs are not uniformly confronted with placental tissues. According to resident tissue, dMφ were categorized into decidua basalis-associated macrophages (decBAMs) and decidua parietalis-associated macrophages (decPAMs) (37) (**Table 2**). The decBAMs (CD163^+^CD206^+^CD11c^hi^HLA-DR^lo^) secreted pregnancy-sustaining factors (IL-10, VEGFA, HMOX1) and promoted Treg induction, aligning with transcriptional profiles of scRNA-seq-defined CD11c^hi^ dMφs. The decPAMs (CD163^+^CD206^+^CD11c^lo^HLA-DR^+^) displayed APC-like activity (phagocytosis, T cell activation), likely maintaining immune surveillance in non-invasion zones.

Above results showed that the dMφs subset were complex and were affected by techniques, markers, and tissue collection strategies. There are also some significant discrepancies regarding the distribution of sepcific dMφs subsets. For example, Kammerer et al. observed that 36.9% of dMφs expressed CD209 (22), whereas Greenbaum et al. reported a significantly higher proporation of CD209^+^dMφs, which accounted for 77% (21). However, Krop et al. demonstrated a contrasting predominance of CD209^-^dMφs (52%) (20). In the trophoblast cell microenvironment from the human first-trimester, IMC showed that dMφs localized proximal to EVT were two HLA-DR^-^ subclusters (dMφ1 and dMφ4) (20). However, spatial proteomics and transcriptomics showed that HLA-DR^+^Mac2a were detected close to EVT (21).

In conclusion, the in-depth understanding of subsets provides an opportunity to open an avenue for the significance of dMφs during pregnancy.

## 4 Factors influencing dMφs recruitment and residence

DMφs are often detected in close vicinity of invading trophoblasts and in the vessel wall of the actively remodeling vessels (38). However, the factors affecting recruitment and residence of dMφs in these sites have not been fully clarified. Previous reviews described that the constitution of adult tissue Mφs includes long-lived Mφs from yolk sac erythro-myeloid progenitors (EMP) and fetal liver hematopoietic stem cells (HSC) as well as short-lived bone marrow HSCs-derived monocytes (1, 6, 39). So far, the studies of dMφs originated from long-lived Mφs were limited (16). Thus, in this part, we will mainly review the recruitment and residence of dMφs originated from short-lived peripheral monocyte (pMo) cells.

### 4.1 Chemokines and growth factors

**Figure 1:**
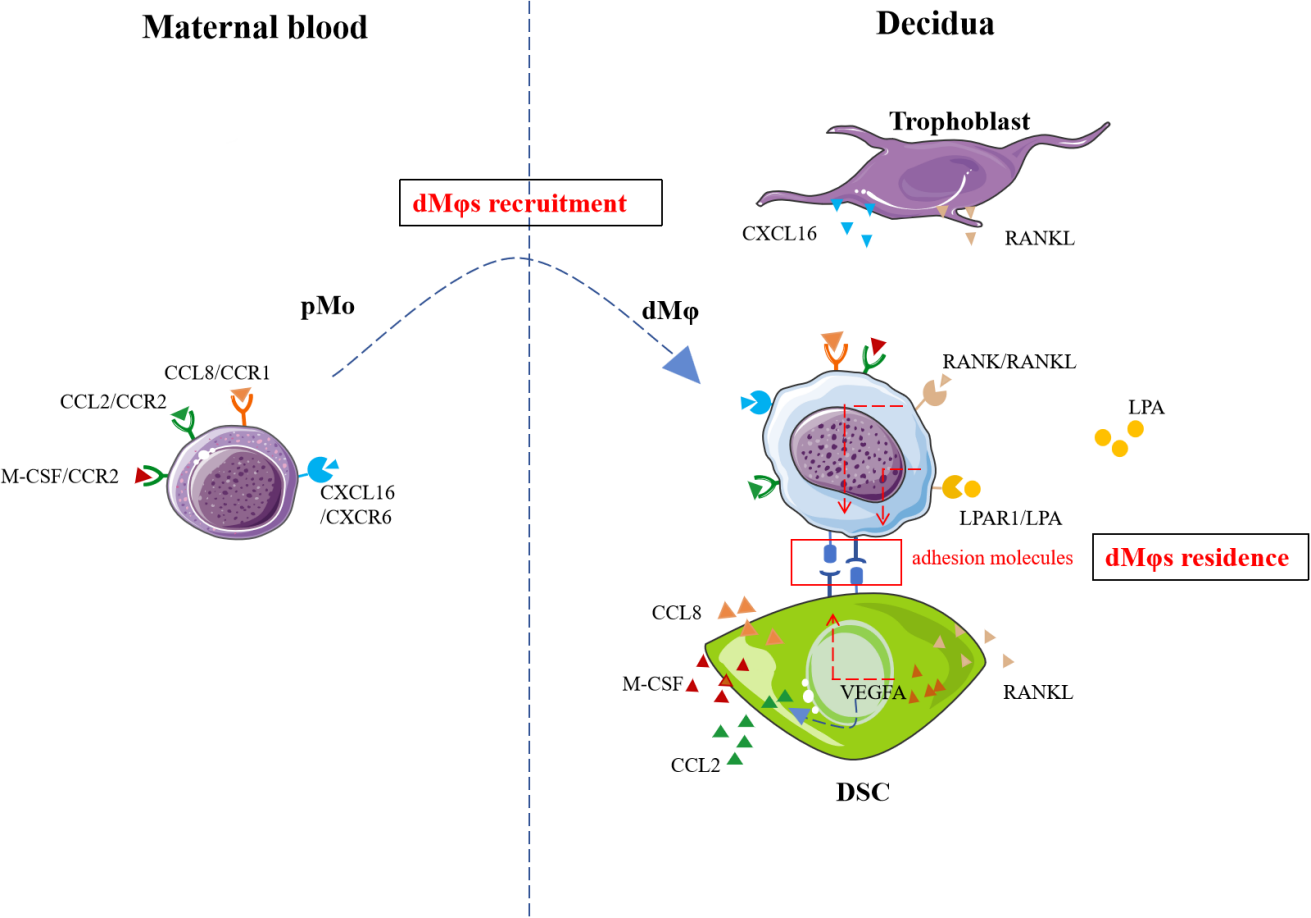
The recruitment and residence mechanisms of dMφs. Recruitment signaling including CXCL16/CXCR6, CCL8/CCR1, CCL2/CCR2, M-CSF/CCR2 and VEGFA promoted the migration of pMo cells to decidua. RANKL/RANK, LPA/LPAR1 and VEGFA facilitate the residence of dMφs. pMo, peripheral blood monocytes; CXCL16, CXC motif chemokine ligand 16; CCL8, CC chemokine ligand 8; CCR1, chemokine-receptor 1; LPA, lysophosphatidic acid; LPAR1, LPA receptors; VEGFA, vascular endothelial growth factor A; RANKL, receptor activator of nuclear factor-kappa B ligand; RANK, receptor activator of nuclear factor-kappa B; M-CSF, macrophage-colony stimulating factor; DSC, decidual stromal cell. The figure was produced by Microsoft Office PowerPoint.

Chemokines, categorized into C, CC, CXC, and CX3C subfamilies based on conserved cysteine motifs, regulate immune cell trafficking through interactions with G-protein-coupled receptors (40). At the maternal-fetal interface, trophoblasts and DSCs secrete multiple chemokines during human early pregnancy (41–46). First-trimester trophoblasts produced CXCL16 (47), which engaged CXCR6 receptors expressed on pMo and dMφs (48). Functional studies confirmed that the CXCL16/CXCR6 axis critically mediated pMo recruitment (48) (**Figure 1**). Notably, CXCR6 expression declined after pMo differentiate into dMφs (87.92%→47.74%) (48), suggesting this signaling may primarily mediate monocyte recruitment rather than post-differentiation retention. Concurrently, DSCs secreted CCL8 and CCL2, which cooperatively enhanced dMφs chemotaxis through CCR1 and CCR2 receptors, respectively (29, 49, 50) (**Figure 1**). In addition, it was confirmed that CCL2 level was regulated by VEGFA (**Figure 1**). For example, VEGFA promoted the secretion of CCL2 from DSCs in hypoxia environment (50). In addition, VEGFA up-regulated the expression of adhesion molecules (ICAM-1, ICAM-5) in DSCs and thus facilitated dMφs anchorage to decidual tissues (50) (**Figure 1**). M-CSF/CCR2 interaction also induced the dMφs recruitment (51) (**Figure 1**).

### 4.2 RANKL-RANK

Liao et al. showed that RANK^+^dMφs from human early pregnancy was the dominating subset with higher adhesion molecules expression (CD29, CD31, CD54, CD62L) (52). The interaction of RANKL secreted by DSCs and RANK on dMφs increased the expression of adhesion molecules on dMφs (**Figure 1**), which in turn allowed dMφs to infiltration into the decidua (52).

### 4.3 Lysophosphatidic acid metabolism

A previous report by microarray data analysis indicated genes PPARγ was highly expressed by CD11c^hi^dMφs from human early pregnancy (13). However, the potential function was unknown. Recently, metabolomics analysis in human dMφs indicated an increased lysophosphatidic acid (LPA) metabolism and high levels of LPA receptors including specific cell-surface G protein coupled receptors LPAR1 and the intracellular receptor PPARγ (53). Yang et al. confirmed that the activation of LPA/LPAR1 or LPA/PPARγ signaling promoted dMφs adhesion to DSCs in a dose-dependent manner by up-regulating adhesion molecules including E-cadherin, E-selectin, L-selectin and integrinαV *in vitro* (**Figure 1**). Mechanistically, this process was mediated through activation of the macroautophagy/autophagy, and further up-regulation of multiple adhesion factors (cadherins and selectins) in a claudin 7-dependent manner (53).

## 5 Factors influencing dMφs polarization and function

Previous findings have consistently shown that the number of M1-like dMφs is higher in women with pregnancy complications such as RSA and PE (54, 55). These observations suggest that a balance between M1/M2-like dMφs is crucial in maintaining decidual homeostasis. Mφs may be shaped by the tissues in which they reside, and they are able to change their functions in response to different microenvironments, forming a broad repertoire of Mφs functions. However, factors involved in M1/M2-like dMφs homeostasis are largely unknown. Recently, advances about regulatory networks underlying dMφs polarization at the human maternal-fetal interface have been achieved, which will provide novel opportunities for manipulating various pregnancy complications.

### 5.1 Cytokines

**Figure 2:**
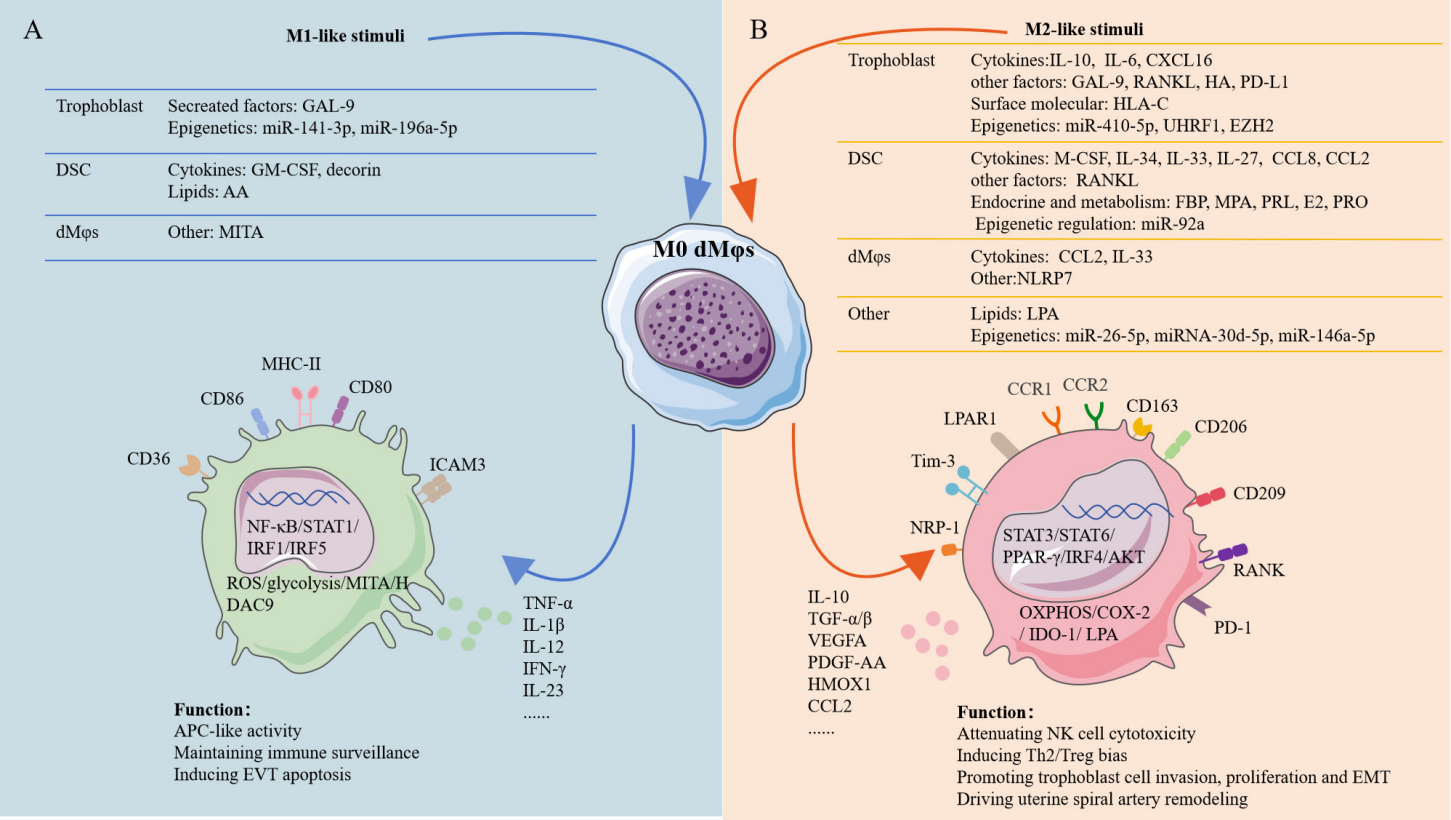
The factors shaping dMφs polarization at the human early maternal-fetal interface. Stimuli of M1-like dMφs and M2-like dMφs were mainly originated from fetal trophoblast, maternal DSCs an dMφs. **(A)** Transcription factors in M1-like dMφs mainly involve NF-κB, STAT1, IRF1 and IRF5. M1-like dMφs phenotype: high expression of CD80, CD86, MHC-II, CD36 and ICAM-3 in cell surface as well as ROS, MITA and HDAC9 inside the cell; secreting high level of TNF-α, IL-1β, IL-12, IFN-γ and IL-23; activated glycolysis. **(B)** Transcription factors in M2-like dMφs mainly involve STAT3, STAT6, PPAR-γ and IRF4; M2-lile dMφs phenotype: high expression of CD163, CD209, CD206, RANK, PD-1, CCR1, CCR2, NRP1, Tim-3 and LPAR1 in cell surface as well as COX-2 and IDO-1 inside the cell; secreting high level of IL-10, TGF-β, VEGFA, CCL2 and HMOX1; activated OXPHOS and LPA metabolism. AA, arachidonic acid; PRL; prolactin; HA, hyaluronic acid; FBP, fructose-1,6-bisphosphate; MPA, medroxyprogesterone acetate; E2; estradiol; PRO, progesterone; IRF1, interferon regulatory factor 1; PPAR-γ, peroxisome proliferator-activated receptor gamma; ICAM-3, intercellular adhesion molecule 3; NRP1, neuropilin 1; HMOX1, heme oxygenase 1; COX-2, cyclooxygenase-2; IDO-1, indoleamine 2,3-dioxygenase 1; T-cell immunoglobulin mucin-3 (Tim-3); PDGF, platelet-derived growth factor; EMT, epithelial-to-mesenchymal transition. The figure was produced by Microsoft Office PowerPoint.

Human first-trimester decidual cells secrete many colony-stimulating factors (CSFs), which then acted as potent inducers of Mφs proliferation, differentiation, and activation. The pre-eclamptic decidua contained an excess of both GM-CSF and M-CSF (56, 57). In response to pro-inflammatory stimulation *in vitro*, human first-trimester decidual cells (leukocyte-free) also enhanced GM-CSF and M-CSF expression (56, 57) (**Figure 2**). GM-CSF drove human pMo cells toward an M1-like subtype, while M-CSF polarized human pMo cells toward an M2-like subtype (23) (**Figure 2**). During the pathogenesis of PE, GM-CSF promoted pro-inflammatory M1-like dMφs being the predominant subtype while M-CSF induced immunosuppressive M2-like phenotype serving as a compensatory response to modulate the decidual immune balance (23, 56, 57). IL-34, a second ligand for the M-CSF receptor, was produced by first-trimester DSCs (**Figure 2**). IL-34, *in vitro*, was able to polarize pMo cells toward an M2-like phenotype (58).

IL-10 was expressed by fetal trophoblasts at the human maternal-fetal interface, increasing from 7.33 pg/mL at 5 weeks to 9.99 pg/mL at≥9 weeks of gestation (23). IL-10 and M-CSF both promoted dMφs polarization with higher CD14, CD163, CD206 and CD209 expression and decreased ICAM-3 expression *in vitro (* 23) (**Figure 2**). Compared with M-CSF, IL-10 was more potential inducer of M2-like dMφs. M-CSF plus IL-10 induced Mφs that displayed the closest phenotype to dMφs. Unexpectedly, classical Th2 cytokines (IL-4, IL-13), orchestrating the classic M2 polarization, were not able to promote the polarization of M2-like dMφs *in vitro (* 23). Recently, Wang et al. further demonstrated that M2-like dMφs induced by IL-10 were linked with OXPHOS changes in mice (59) (**Figure 2B**). However, the related mechanism in human remains needs to be explored.

IL-6 is a multifunctional cytokine, which promoted M2 polarization in solid tumors and inflammatory environments (60, 61). IL-6 was recently found as a potential driver for M2-like dMφs in human pregnancy (62). Reduced jupiter microtubule-associated homolog 2 (JPT2) in RSA patients, correlated with down-regulated M1-like dMφs. Mechanistically, JPT2-deficient trophoblasts exhibited impaired IL-6 secretion, triggering M1 polarization and ROS overproduction—reversed by IL-6 supplementation (62) (**Figure 2A**). IL-27 interacts with a heterodimeric receptor composed of IL-27Rα and gp130 (63), presenting a wide spectrum of different functions ranging from promoting or curbing inflammatory diseases, cancers, and viral infections (64, 65). At the human early pregnancy, IL-27 from DSCs interacted with IL-27R on dMφs induced the COX-2^+^dMφs presenting an M2-like phenotype (31) (**Figure 2B**). Consistently, lower IL-27 in DSCs and a lower percentage of M2-like COX-2^+^dMφs from RSA patients were detected. However, excessive COX-2 in dMφs induced by excessive arachidonic acid (AA) metabolism from RSA patients leaded to severe inflammation by accumulating PGE2 and IL-1β (34).

IL-33, a member of the IL-1 family, is widely expressed under normal physiological conditions. IL-33 activates both the innate and adaptive immune systems through binding to the ST2 receptor. IL-33 and its orphan receptor ST2 were found to be co-expressed by DSCs and dMφs in human first-trimester pregnancy (**Figure 2** **) (** 66). In RSA patients, decreased IL-33 was observed in DSCs and dMφs. *In vitro*, inhibited IL-33/ST2 signaling drove classical M1-like dMφs polarization (66).

### 5.2 Chemokines

In addition to being involved in cell recruitment and residence of dMφs, chemokines also play a pivotal role in dMφs polarization.

Trophoblasts from human early pregnancy secreted substantial quantities of CXCL16, with CXCR6 serving as its exclusive receptor (47, 48). This trophoblast-derived chemokine polarized primary human pMo cells toward an immunoregulatory phenotype, up-regulating M2-associated markers (CD163, CD206, IL-10) while suppressing M1-related molecules (CD80, CD86, IL-12) (**Figure 2**). Consequently, this phenotype shift reduced IL-15 production, thereby attenuating NK cell cytotoxicity (47).

CCR1^+^dMφs in human early pregnancy exhibited a significant M2-like phenotype. Furthermore, DSCs from human early pregnancy exhibited elevated expression of CCL8 (29), which functioned as cognate ligand for CCR1. Recently, elevated CCL8 from DSCs was confirmed as a regulator of CCR1^+^dMφs as indicated that CCL8 recruited peripheral CCR1^+^pMo cells, educated CCR1^+^pMo into CCR1^+^dMφs-like immunosuppressive subsets, and reinforced the CCR1^+^dMφs- exerted modulation of trophoblasts *in vitro* (29) (**Figure 2**). In RSA patients, CCL8 expression in DSCs was decreased and epithelial-to-mesenchymal transition (EMT) of trophoblast was defective.

At the human first-trimester decidua, expression of CCL2 was mainly detected in dMφs and DSCs (3). A previous finding that dMφs could be divided into three subsets based on CCR2 and CD11c showed that CCL2/CCR2 axis was essential for dMφs subpopulations (36). Wei et al. found that the anti-inflammatory status of dMφs was dependent on the CCL2/CCR2 signaling because the CCR2 inhibitor decreased CD163 expression of dMφs, whereas CD80 and CD86 expression were unaffected (3) (**Figure 2**). Therefore, CCL2 might influence the immune status of dMφs at the maternal-fetal interface in an autocrine and paracrine manner.

### 5.3 Extracellular matrix

Hyaluronan (HA) is found ubiquitously in the extracellular matrix (ECM) of all mammalian tissues. Beyond its well-established structural contributions to ECM organization and tissue homeostasis, accumulating experimental evidence demonstrates that HA actively participates in immunomodulatory processes (67, 68). CD44 is the principal receptor of HA (69), and HA/CD44 signaling has long been known to play a role in immune regulation. In human early pregnancy, primary trophoblasts could secreted high molecular weight HA (HMW-HA) continuously and about 80% of dMφs express CD44 (70). Wang et al. confirmed that treatment of dMφs from human early pregnancies with HMW-HA significantly up-regulated M2-associated markers while down-regulated M1-associated markers through CD44-mediated activation of the PI3K/AKT and STAT3/STAT6 signaling pathways (70) (**Figure 2B**).

Decorin is a member of proteoglycan family and involved in regulating collagen fibrillogenesis (71). In human early pregnancy, decorin was expressed by DSCs and significantly up-regulated in DSCs from RSA patients (72). Aberrant decorin level was related to various pregnancy complications (73). A positive correlation between decorin content and the proportion of M1-like dMφs was also observed in the decidua of early normal pregnant women (72). In murine Mφs, decorin treatment induced M1-like Mφs polarization, which was related to enhanced glycolysis, increased mitochondrial membrane potential and intracellular ROS levels (**Figure 2A**).

### 5.4 Immune checkpoints

Multiple immune checkpoints dynamically regulated dMφs polarization such as galectin-9 (Gal-9)/Tim-3 and PD-1/PD-L1 signaling (**Figure 2**). As mentioned above, Tim-3^+^dMφs demonstrated pro-angiogenic capacity and immunomodulatory activity (25, 26). Higher Tim-3 expression on dMφs was dependent on HLA-C on trophoblast during normal pregnancy (25) (**Figure 2**). Consistently, Gal-9/Tim-3 signaling alleviated inflammation by inducing M2-like polarization in rodent models of PE (74) (**Figure 2**). However, Gal-9/CD44 signaling promoted M1-like polarization associated with vascular dysfunction and PE risk in human pregnancy (55) (**Figure 2**). This functional dichotomy suggested receptor-dependent modulation of Gal-9. The PD-1/PD-L1 signaling further reinforced polarization homeostasis, where down-regulated PD-1 expression on dMφs and attenuated PD-L1 expression in placental villous tissues in RSA correlated with M1-like dMφs dominance (54). Experimental blockade studies confirmed PD-1 signaling inhibition critically promoted M1-like dMφs polarization by enhancing glycolysis and IRF5 activation (54) (**Figure 2A**).

### 5.5 Other factors

The RANK/RANKL signaling in osteoclasts regulates bone resorption *via* activating NF-κB pathway. However, the RANK/RANKL signaling predominantly drove M2-like polarization *via* AKT/STAT6/IRF4 signaling (35) (**Figure 2B**). When stimulated with RANKL, RANK^+^dMφs from human early pregnancy promoted Th2 bias but had no effect on decidual Treg cell differentiation (35).

### 5.6 Endocrine and metabolism

Mφs metabolic activity is an essential factor regulating their polarization and function (75, 76). Compared with human pMo cells, human dMφs from early pregnancy were significantly rich in LPA metabolism and expressed higher LPA receptor including specific cell-surface G protein coupled receptors LPAR1 and the intracellular receptor PPARγ (53) (**Figure 2B**). In pregnant mouse model, LPA deficiency promoted M1 polarization (53). Further research about whether LPA was involved in M2-like polarization of human dMφs is needed.

In the classical M1/M2 paradigm, CD36-dependent triglyceride transport is indispensable for M2 Mφs polarization. Contrasting this paradigm, CD36 paradoxically marked pro-inflammatory M1-like dMφs during early pregnancy, a functional shift mechanistically linked to its AA transport activity. In RSA patients, excessive AA accumulation was frequently observed in both DSCs and dMφs (34). Excessive accumulated AA was transferred from DSCs to dMφs *via* CD36 on dMφs, which excessively activated COX-2/PGE2/IL-1β signaling and promoted M1-like polarization *in vitro (* 34) (**Figure 2A**). While in normal pregnancy, higher prolactin (PRL) from human DSCs was detected, which down-regulated CD36 expression in human dMφs, inhibiting lipid influx and the inflammatory phenotype of dMφs (34). This bidirectional regulation highlighted how microenvironmental cues reprogrammed CD36 functionality.

In addition to lipid accumulation, fructose-1,6-bisphosphate (FBP) was also accumulated in DSCs during human early pregnancy (31). FBP is considered responsible for sustaining glycolysis and increasing ATP production, eventually accelerating the decidualization. As mentioned above, IL-27 was identified as a new inducer of M2-like dMφs during early pregnancy. Further, IL-27 secreted by human DSCs was mainly promoted by FBP (**Figure 2**). Previously, it was considered that neither progesterone (PRO) nor estradiol (E2) showed any effects on the differentiation of M2-like dMφs induced from isolated CD14^+^pMo cells (23). However, PRO was found to promote the enrichment of FBP and IL-27 in DSCs isolated from first-trimester decidua (31) (**Figure 2**). Above results indicated the indirect role of FBP and PRO on regulating M2-like dMφs polarization. IDO^+^dMφ displayed a M2-like dominate phenotype during early pregnancy (32). Expression of IDO was increased remarkably after treatment dMφs with medroxyprogesterone acetate (MPA) or E2 (32) (**Figure 2**), suggesting that the endocrine environment contributed to the high level of IDO in dMφs during early pregnancy.

### 5.7 Post-transcriptional and epigenetic regulation

Post-transcriptional and epigenetic regulation are key mechanisms controlling gene expression. Post-transcriptional regulation is partly mediated by miRNAs, while epigenetic regulation—primarily involving DNA methylation (and active demethylation via hydroxymethylation intermediates) and histone modifications—can produce heritable phenotypic changes without altering the DNA sequence. Emerging evidence highlights their critical role in maintaining decidual immune homeostasis during early gestation. For instance, trophoblasts modulated dMφs polarization through miRNA-mediated pathways: miR-410-5p enhanced M2-like dMφs polarization by suppressing STAT1 (77) (**Figure 2**). Notably, miR-410-5p expression was significantly reduced in RSA patients compared to normal pregnancies (77) (**Figure 2**). Conversely, hypoxia-preconditioned trophoblasts (mimicking PE) and RSA-derived trophoblasts exhibited elevated miR-141-3p (78) and miR-196a-5p (79) levels, respectively. These miRNAs drove M1-like dMφs polarization *via* NF-κB pathway activation (78, 79) (**Figure 2A**). Additional miRNAs contributed to dMφs polarization: miR-92a (DSC-derived) (80), miR-26-5p (seminal plasma-derived) (81), and placental miRNAs of unclear origin (miR-146a-5p, miR-30d-5p) (82, 83) promoted M2-like dMφs polarization (**Figure 2**). Mechanistically, miR-30d-5p targeted histone deacetylase 9 (HDAC9), whose knockdown enhanced M2-like dMφs polarization (83).

Enhancer of zeste homolog 2 (EZH2) is a histone methyltransferase, which mediates the transcriptional silencing of target genes *via* H3K27me3 (84). Ubiquitin like with PHD and ring finger domains 1 (UHRF1) maintains DNA methylation status (85). Both EZH2 and UHRF1 were expressed by trophoblasts and down-regulated in RSA patients. The conditioned medium from EZH2 or UHRF1 knockdown trophoblasts both promoted M1-like dMφs polarization, indicating an indirect effect (86, 87).

### 5.8 NLRP7 and pyroptosis

The NOD-like receptor (NLR) family, a critical class of pattern recognition receptors (PRRs), typically mediates inflammasome assembly (NLRP1, NLRP3), pro-inflammatory cytokine release (IL-1β, IL-18), and pyroptosis—hallmarks of M1 Mφs activation (88). However, the role of NLRP7 seems different in dMφs. Unlike canonical NLRs, NLRP7 demonstrated preferential expression in M2-like dMφs compared to M1-like dMφs in the human first-trimester endometrial tissues (89). Functional studies revealed NLRP7 overexpression suppressed M1 markers while enhancing M2 polarization signatures.

Pyroptosis, a marker of M1 Mφs, was also regulated by mitochondrial adaptor protein MITA (90). Liu et al. showed that M1-like dMφs maintained elevated MITA levels to promote pyroptosis, while M2-like dMφs employed TRIM38 mediated K48-linked ubiquitination to degrade MITA, effectively suppressing pyroptosis (91). This polarization-dependent mechanism was clinically validated in RSA cases, where decidual tissues exhibited enhanced pyroptotic markers, higher MITA expression and impaired M2-like dMφs polarization (91).

## 6 Conclusion

The balance between pro-inflammatory (M1-like) and anti-inflammatory (M2-like) dMφs subsets emerges as a linchpin for maintaining immune homeostasis, with perturbations in this equilibrium linked to adverse pregnancy outcomes. This review systematically characterized the unique characteristics of dMφs subsets—distinct from classical Mφ polarization characteristics—and explored their potential utility in clinical diagnostics for distinguishing M1-like/M2-like dMφs subsets.

The recruitment mechanisms and factors controlling dMφs polarization offer actionable therapeutic targets. For instance, enhancing M2-like polarization *via* STAT3 and STAT6 activation or modulating placental-derived signals could mitigate excessive inflammation in RSA or PE. Furthermore, interventions targeting dMφs recruitment (CCR2/CCL2 signaling) or tissue-residency programs could restore decidual immune balance.

By bridging mechanistic insights with clinical translation, this synthesis underscores that precision modulation of dMφs dynamics—through small molecules, biologics, or cell-based therapies—holds transformative potential for treating pregnancy complications. Future research should prioritize validating these targets in preclinical models and developing biomarker-driven strategies to tailor interventions, ultimately advancing personalized care for gestational disorders.
